# Advanced Methods for the Characterization of Supramolecular Hydrogels

**DOI:** 10.3390/gels7040158

**Published:** 2021-09-29

**Authors:** Bridget R. Denzer, Rachel J. Kulchar, Richard B. Huang, Jennifer Patterson

**Affiliations:** 1 Department of Chemical and Biological Engineering, Princeton University, Princeton, NJ 08544, USA; denzer@princeton.edu (B.R.D.); rbhuang@princeton.edu (R.B.H.); 2 Department of Chemistry, Princeton University, Princeton, NJ 08544, USA; rkulchar@princeton.edu; 3 Biomaterials and Regenerative Medicine Group, IMDEA Materials Institute, Getafe, 28906 Madrid, Spain; 4 Independent Consultant, 3000 Leuven, Belgium

**Keywords:** spectroscopy, diffraction, microscopy, rheology, biomaterials

## Abstract

With the increased research on supramolecular hydrogels, many spectroscopic, diffraction, microscopic, and rheological techniques have been employed to better understand and characterize the material properties of these hydrogels. Specifically, spectroscopic methods are used to characterize the structure of supramolecular hydrogels on the atomic and molecular scales. Diffraction techniques rely on measurements of crystallinity and help in analyzing the structure of supramolecular hydrogels, whereas microscopy allows researchers to inspect these hydrogels at high resolution and acquire a deeper understanding of the morphology and structure of the materials. Furthermore, mechanical characterization is also important for the application of supramolecular hydrogels in different fields. This can be achieved through atomic force microscopy measurements where a probe interacts with the surface of the material. Additionally, rheological characterization can investigate the stiffness as well as the shear-thinning and self-healing properties of the hydrogels. Further, mechanical and surface characterization can be performed by micro-rheology, dynamic light scattering, and tribology methods, among others. In this review, we highlight state-of-the-art techniques for these different characterization methods, focusing on examples where they have been applied to supramolecular hydrogels, and we also provide future directions for research on the various strategies used to analyze this promising type of material.

## 1. Introduction

Gels are three-dimensional (3D) materials that display solid-like viscoelastic properties, and hydrogels are water-based gels, as indicated by the prefix “hydro” meaning water. For classifying gels based on their structures, four different categories were presented by Paul Flory in 1974, specifically (i) ordered gels composed of lamellar networks, (ii) covalent polymer gels, (iii) less-ordered physical polymer gels, and (iv) gels composed of polymers or fibers [1]. One sub-type of hydrogel, based on supramolecular interactions, has become increasingly utilized in many biomaterial applications as well as in the areas of energy, optoelectronics, and water purification, among others [2]. Supramolecular chemistry was defined as “the chemistry of molecular assemblies and of the intermolecular bond” by Jean-Marie Lehn who won a Nobel Prize for his work in supramolecular chemistry in 1987. More recently, it has been referred to as “non-molecular chemistry” by many [3]. Unlike covalently crosslinked hydrogels, supramolecular hydrogels are based on non-covalent bonding such as hydrogen bonding, electrostatic interactions, and hydrophobic interactions between the molecules that build up the hydrogels [4,5]. The nature of these self-assembled network structures and interactions allows supramolecular hydrogels to be formed through reversible mechanisms, allowing them to transition between gel and solution states [4,6,7]. In this review, we follow the precedent of Christoff-Tempesta et al. in classifying supramolecular hydrogels as those that satisfy category (iv) of the Flory definition, mainly self-assembled structures based on low-molecular-weight gelators, as well as hydrogels that follow categories (i) and (iii) as long as they exhibit non-covalent bonding [2].

Improving our understanding of the material properties of supramolecular hydrogels is crucial to the continued advancement of their use in various fields. Developments in characterization methods enable the more sophisticated analysis of these material properties, yielding information on, for example, the mechanisms of hydrogel formation or how the formation of non-covalent and covalent bonds influences mechanical properties [8]. This review provides an updated overview of characterization methods for supramolecular hydrogels, covering how they have been traditionally applied as well as more recent advancements such as saturation transfer difference (STD) nuclear magnetic resonance (NMR), surface-enhanced Raman scattering (SERS), cryogenic electron microscopy (cryo-EM), super-resolution microscopy (SRM), and micro-rheology techniques. While a main emphasis is on supramolecular hydrogels because of their unique properties and behavior, many of these methods are also relevant for the characterization of hydrogels in general as well as of other supramolecular gels (organogels [9], metallogels [10], etc.).

**Figure 1:**
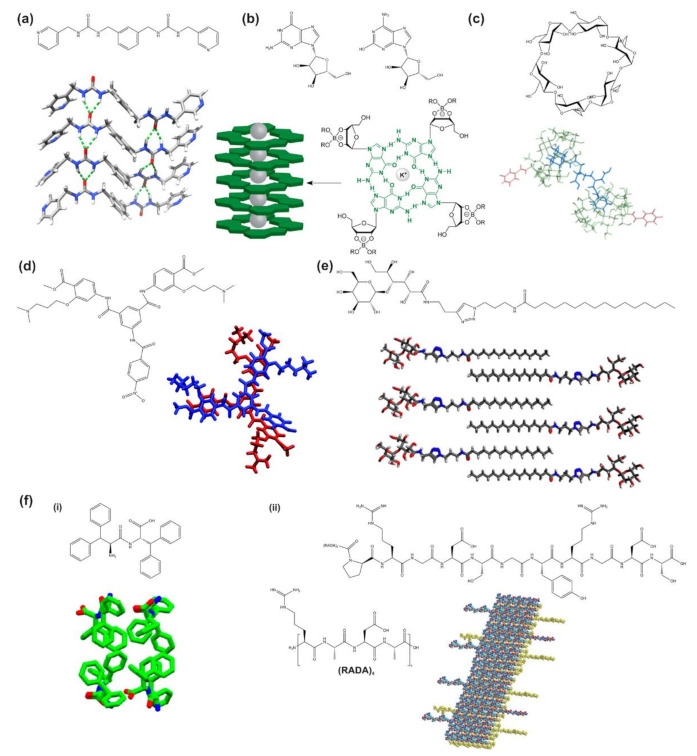
Schematics of representative molecules from different classes of supramolecular hydrogelators showing the chemical structures and how they are hypothetically interacting to self-assemble, including (**a**) bis-urea derivatives, (**b**) nucleosides and nucleoside derivatives, (**c**) cavity-bearing molecules, (**d**) polyaromatic compounds, (**e**) saccharide derivatives, and (**f**) amino acids and peptides and their derivatives. The schematics of the self-assembled structures are reproduced (**a**) from [14] with permission, ©2019—Royal Society of Chemistry; (**b**) from [15] under the terms of a CC-BY license, ©2014—American Chemical Society; (**c**) from [16] under the terms of the Creative Commons Attribution License, ©2014—the authors and published by Beilstein-Institut; (**d**) from [17] with permission, ©2019—Royal Society of Chemistry; (**e**) from [18] with permission, ©2017—Elsevier; and (**f**) from (**i**) [19] under the terms of a CC-BY license, ©2020—American Chemical Society and (**ii**) [20] under the terms of the Creative Commons Attribution License, ©2007—the authors and published by PLoS ONE.

Herein, we focus on supramolecular hydrogels that are formed from small molecules including bis-urea derivatives, nucleosides and nucleoside derivatives, polyaromatic compounds, cavity-bearing molecules, saccharide derivatives, and amino acids and peptides and their derivatives (Figure 1) [5]. These small molecules, also known as hydrogelators, undergo self-assembly into supramolecular hydrogels that hold together due to the non-covalent bonding between the molecules. Peptide-based supramolecular hydrogels utilize amino acids themselves, short peptides, or peptide amphiphiles as the hydrogelator molecules, which is advantageous for biological applications due to the biocompatibility of these molecules [11]. Another main type of supramolecular hydrogel is based on host–guest interactions. The host molecule, typically cyclodextrin or cucurbituril, has an opening space where the guest molecule can fit into and bind through non-covalent or hydrophobic interactions [12]. Supramolecular hydrogels can be formed through inserting the host–guest complexes into polymer chains, or by using the host–guest molecules as monomers that polymerize together when exposed to ultraviolet light, as Wang et al. demonstrated [13]. Supramolecular hydrogels can also be formed from small organic molecules, such as those including urea or pyridine groups, as well as from nucleobase and saccharide derivatives, as extensively reviewed [5].

Specifically, this review investigates various methods suitable for the characterization of supramolecular hydrogels, beginning with spectroscopy, which includes NMR spectroscopy, absorption spectroscopy, Raman spectroscopy, and circular dichroism (CD), which help elucidate the composition and structure of the hydrogels. The next section focuses on diffraction, with X-ray diffraction (XRD) being most prominently used in the field, although other methods such as neutron diffraction also exist. The third section covers microscopy, including traditional transmission and scanning electron microscopies, while also including methods such as cryo-EM and fluorescence and super-resolution microscopies. This section also includes recent advancements in scanning probe microscopy (SPM), specifically atomic force microscopy (AFM), which can be used to image hydrogels at higher resolution as well as to obtain information about their mechanical properties. The final section concludes with methods that are also important for the characterization of the mechanical and surface properties of these materials, such as rheology, micro-rheology, dynamic light scattering (DLS), and tribology. Throughout, this review includes a perspective on the combination of experimental approaches with computational modelling and simulations, an important tool in modern-day research, and thus we provide an outlook for their expanded use in future studies. Finally, we note that most techniques for the characterization of soft materials can be utilized in general with gels prepared in a variety of solvents (e.g., hydrogels, organogels) and with supramolecular hydrogels in particular. While we sometimes rely on studies with a broader focus to be able to explain the techniques, in each section we include examples of how these methods have been used with supramolecular hydrogels to specifically illustrate the relevant information that can be obtained from this characterization.

## 2. Spectroscopy

Spectroscopic methods, such as the technologies of NMR, ultraviolet–visible (UV–vis), Fourier-transform infrared (FT-IR), and Raman spectroscopy as well as CD, are frequently used in the characterization of supramolecular hydrogels to infer atomic- and molecular-level structural and morphological data. These experiments are able to analyze gel-solution interface dynamics and binding processes, which are important to characterize the formation and stability of supramolecular hydrogels. Host–guest chemistries, which relate to the ability of hydrogels to physically capture, rather than chemically capture via covalent bonding, other guest molecules within themselves, can also be examined. These methods are generally useful for many types of hydrogels, although the chirality of supramolecular assemblies is often of concern when considering supramolecular hydrogels in particular. Chirality can be important in understanding and designing for material functionality and is most often investigated with CD spectroscopy [21,22]. Additionally, DLS and diffusing-wave spectroscopy (DWS) are other spectroscopic techniques useful for characterizing supramolecular hydrogels, but these are discussed in detail in Section 5.3 in context to their application to micro-rheology studies.

### 2.1. NMR Spectroscopy

NMR spectroscopy is a popular, non-invasive characterization technique from which a lot of information can be derived, when applied correctly [23]. A fundamental application of NMR spectroscopy is to determine the presence of certain species and functional groups in analytes, and comparing multiple NMR spectra after changing an experimental condition allows one to infer changes to molecular structure. Of relevance to supramolecular chemistry, NMR applied to solutions is known as solution-state NMR and provides information on solution composition. Commonly, the hydrogen nuclei are studied using ^1^H NMR spectroscopy in the solution-state; however, gelation and the concomitant reduction in mobility is a limitation to this particular technique [23]. NMR can, nevertheless, be used to monitor the gelation process of supramolecular hydrogels, and applying NMR to the hydrogel after gelation involves solid-state NMR, which can observe hydrogel structure and composition and infer conformational data and interactions with local chemical environments [24].

**Figure 2:**
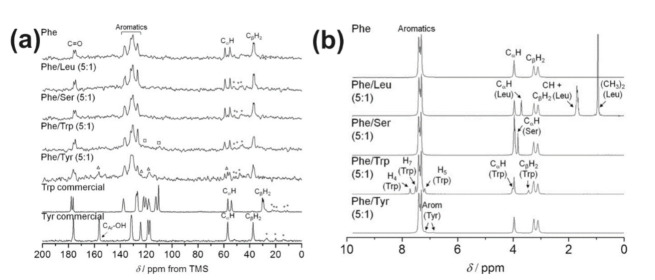
(**a**) Multiple solid-state NMR spectra of phenylalanine hydrogels, two-component hydrogels, and starting materials arranged for comparison. (**b**) Solution-state NMR spectra of phenylalanine hydrogels and two-component hydrogels with different amino acids added. Abbreviations: phenylalanine (Phe), leucine (Leu), serine (Ser), tryptophan (Trp), and tyrosine (Tyr). Reproduced under the terms of the Creative Commons Attribution License from [25]. ©2017 The Authors. Published by Wiley-VCH Verlag GmbH & Co. KGaA.

Solid-state NMR is a useful tool for investigating the molecular structure of hydrogels in a solid-like gel phase, and it involves methods such as magic-angle spinning (MAS) NMR. Ramalhete et al. utilized ^1^H-^13^C cross-polarization MAS NMR in their investigation into the formation process of different amino acid-based hydrogels. In this study, phenylalanine was combined with one of tyrosine, leucine, serine, or tryptophan to form two-component solutions to undergo gelation, and the resulting materials were characterized via solid-state and solution-state NMR. Solid-state NMR was used on the mixed hydrogels as well as a single-component phenylalanine hydrogel and solid powder forms of tryptophan and tyrosine to obtain spectra that could be compared to determine the extent that each of the additional amino acids participated in forming the rigid parts of the hydrogel (Figure 2a). Rigid sections of the hydrogel exhibited additional coupling between the ^1^H and ^13^C with peaks in the NMR spectra that could be attributed to phenylalanine, tyrosine, and tryptophan, indicating their incorporation in the rigid hydrogel network while leucine and serine remained mobile [25]. Reddy et al. used ^7^Li, ^11^B, ^23^Na, ^39^K, and ^133^Cs MAS NMR in a study investigating the process in which supramolecular guanosine-borate hydrogels take up small molecules. In one experiment adding adenine molecules to guanosine-quartet Na^+^ borate hydrogels, the shifting of Na^+^ peaks upon addition of adenine indicated that a change in distribution of Na^+^ ions took place, leading the researchers to conclude that the adenine resided in the pores instead of the framework of the hydrogel. Interestingly, density functional theory (DFT) calculations were also performed to complement the experimental ^11^B NMR data by optimizing the structure of the guanosine-borate monoester and diesters and calculating ^11^B NMR shieldings to correlate with the experimentally measured ^11^B shifts. These showed that the *cis* diester form was the preferred one for gelation [26]. This expanded on prior work by Peters et al., who used solid-state ^11^B MAS NMR to characterize the guanosine-borate hydrogels. Solid-state NMR was utilized to differentiate between two hydrogels that contained 2 wt.% of either K^+^ or Cs^+^ ions. The spectra for both hydrogels exhibited two peaks due to the presence of borate diesters, and one peak corresponded to the sol phase while the other corresponded to the gel phase. These two peaks were separated in the hydrogel with the Cs^+^ ions but overlapped in the K^+^ hydrogel, indicating that the latter hydrogel had more of the borate diester in the gel phase [15]. Thus, solid-state NMR is a useful characterization technique for understanding the chemical composition and structure of supramolecular hydrogels.

Solution-state NMR and variable-temperature NMR are useful for investigating the composition of hydrogel precursor solutions before they have gelled into a solid, and thus they can help in analyzing the gelation process. Variable-temperature NMR is essentially NMR that is performed at different temperatures, and it can be considered solution-state NMR when the temperature is high enough to turn the hydrogel into a solution phase. Solution-state NMR was also used during the experiment by Ramalhete et al. in which all of the supramolecular hydrogels were heated into solution and left to cool and reform into hydrogels. The compositions of the solutions were monitored with solution-state ^1^H NMR throughout the cooling time, providing information on which molecules had been incorporated into the hydrogels and at what rates this occurred during the gelation process. For instance, by examining the intensity of the solution-state NMR peaks over time for a hydrogel made of L-phenylalanine, the researchers were able to determine that about 40% of these molecules were incorporated in the rigid fiber structures in the hydrogel. Additionally, for a hydrogel made up of a 5:1 L-phenylalanine/L-serine mixture, the increasing intensities of the peaks (Figure 2b) indicated that less L-phenylalanine was absorbed into the hydrogel and more was in the solution phase when L-serine was present [25]. Similarly, in a study by Rutgeerts et al., variable-temperature NMR was used to investigate the gelation transition of supramolecular hydrogels made from a bis-urea derivative. The researchers performed NMR on the sample at temperatures between 30 and 90 °C, with increments of 10 °C. As the temperature increased and the hydrogel began transitioning from a gel to a solution, the NMR peaks narrowed and also shifted slightly upfield [14], which is consistent with increased mobility of the protons [23]. Thus, solution-state NMR is a helpful technique for analyzing supramolecular hydrogel compositions, and it can also be used in conjunction with solid-state NMR.

More recent developments of NMR techniques have centered around improving methodologies to understand molecular-level interactions at interfaces. STD NMR, a solution-state technique, has been demonstrated to be useful for supramolecular hydrogel characterization in multiple studies [25,27]. Frequently used in biology to study ligands and proteins in rapid-exchange equilibrium, STD NMR is able to identify those ligands that bind most effectively to a protein and indicate the relative strength of binding [28]. For hydrogels, it can be used to observe exchange phenomena between the hydrogel network and solution phase as well as to analyze ligand binding to substrates in the gels. Ramalhete et al. used this approach when they monitored the sol-gel exchanges as their hydrogels were heated back into solution. By comparing the STD NMR results of the two-component hydrogels to that of the single-component phenylalanine hydrogel, they were able to determine how well each of the additional amino acids was bound to the network and the strength of that binding relative to phenylalanine. Tryptophan and tyrosine were found to bind to the resultant two-component hydrogel more effectively than phenylalanine, while leucine and serine had weak or no binding to the hydrogel network [25]. STD NMR and other techniques were combined in an overall experimental design to classify surface properties of self-assembled peptide hydrogel fibers developed by Wallace et al. using a selected set of probing molecules that reacted to particular surface chemistries, such as surface charge from functional groups and hydrophobicity. Along with STD NMR, residual quadrupolar couplings (RQCs) were used, and they manifest in NMR spectra as a peak that splits into two. RQCs appear in part from anisotropic properties of the gel being tested, and like STD NMR, they have been used to identify which probing molecules most readily react with a gel surface and to measure the relative extent to which they interact. Hydrogels were formed from N-functionalized dipeptides in three separate ways, through changing pH, adding salt, or switching the solvent, and the surface chemistries of the final products differed. Using this set of methods, the researchers were able to determine which among the three surface chemistries had the most negative charge and how each formation method influenced the final surface chemistry even though the composition of the hydrogel itself was the same N-functionalized dipeptide across the three methods. The authors were also able to conclude whether the presence of certain molecules, such as benzylammonium ions, when diffused into hydrogels would significantly alter the gelation process [27].

### 2.2. Absorption Spectroscopy

Absorption spectroscopic techniques shine particular wavelengths of light at a sample and measure the absorbance to determine the type of molecule in the sample as well as its concentration using the Beer–Lambert Law [29,30]. The two most common absorption spectroscopic techniques are UV–vis and FT-IR. UV–vis spectroscopy shines various wavelengths of light in the ultraviolet and visible spectrum through a solution of an analyte and measures the absorbance. Thus, similar to NMR, comparing UV–vis spectra before and after changing a solution variable provides evidence of changes in the composition, concentration, or chemical environment. For example, π-conjugated hydrogelators absorb in the visible region, and absorption spectroscopy of a self-assembling quinquethiophene-oligopeptide derivative showed that the absorbance maximum did not change with a change in solvent but that the peak shape (broadening, shoulder formation) did change, indicating the formation of aggregates [30]. Reaction kinetics can also be tracked if absorbance is affected, such as for hydrogels exhibiting enzymatic activity [31] or during isomerization reactions. For instance, Wang et al. noticed the light-sensitivity of a conductive supramolecular hydrogel formed from azobenzene and α-cyclodextrin (α-CD) related to a *trans*-*cis* isomerization of the azobenzene. Under 365 nm light, a higher-resistance, lower-conductivity form of the hydrogel was formed, while under 420 nm light, lower resistance was achieved. UV–vis spectroscopy was performed on the hydrogel initially and after being irradiated with either 365 nm or 420 nm light, and the absorbance spectra were compared. The differences between the absorption spectra were identified as providing evidence of the isomerization reaction [29]. Thus, UV–vis spectra can be compared more generally as solution or hydrogel chemistries change, to confirm structural changes of the relevant molecules [29,32].

FT-IR spectroscopy is similar in principle to UV–vis spectroscopy, although it is performed using infrared light. Non-FT infrared spectroscopy may refer to dispersive techniques involving measuring the absorbance of light of a single wavelength at time, whereas FT-IR specifically infers the absorbance spectrum by applying the Fourier transform procedure to absorbance measurements of multiple wavelengths of light at once. Certain stretching vibrations on FT-IR spectra correspond to particular functional groups, allowing the method to characterize the atomic structure of the hydrogel or solution or otherwise confirm its composition. For supramolecular hydrogel characterization, by comparing the differences in the FT-IR spectra of solution and gel forms and analyzing what bonds have appeared and disappeared, it becomes possible to infer the driving force of hydrogelation [33,34]. Wu et al. demonstrated this in their development of a dexamethasone sodium phosphate (Dex) hydrogel that was crosslinked with calcium ions for drug delivery. FT-IR was performed on Dex in dry powder form and then on the Dex hydrogel, and their spectra were compared. The differences were pinpointed as relating to the phosphate group in Dex, and it became possible to infer the phosphate group’s involvement in coordination bonds formed during hydrogelation [34]. Similarly, in a study involving a supramolecular hydrogel based on succinated paclitaxel, changes in FT-IR spectra between the starting compound and the hydrogel indicated that hydrolysis of ester bonds as well as ionization of the carboxylate group were likely involved in hydrogel formation [33]. Finally, while routine FT-IR spectroscopy tends to provide more qualitative information on the bulk material composition, more advanced methodologies, such as attenuated total reflection (ATR) FT-IR, are being developed for more precise measurements, surface characterization, and even imaging of spatial distributions of chemical compounds [35].

### 2.3. Raman Spectroscopy

Raman spectroscopy is another standard, non-invasive technique that beams light through an analyte and relies on the scattering of photons from molecular-level transfers of energy. The scattering is highly specific, allowing this technology to gather atomic and molecular structural data and measure properties such as crystallinity. Raman spectral bands indicate the presence of different functional groups, with a fingerprint region unique to each substance. A common application of this technology was demonstrated in a study investigating supramolecular hydrogels formed by self-assembling graphene oxide, created from the oxidation of graphite, and para-aminosalicylic acid, to confirm a structural change in the material as the graphene underwent oxidation [36]. Additionally, a novel development in improving the scope of Raman spectroscopy for the characterization of supramolecular hydrogels is given by Braun et al. who wished to identify the pK_a_, which is important for many pH-responsive hydrogels, of a histidine residue in a self-assembling peptide hydrogel but identified limitations faced by both solution-state and solid-state NMR when analyzing the supramolecular hydrogels. Instead, the histidine residue was doped with deuterium, making the vibrations detectable by Raman spectroscopy sensitive to the protonation state of the histidine. From concentration-normalized Raman difference spectra measured at different pH, the pK_a_ of the compound could be precisely determined [37]. In this sense, Raman spectroscopy is a useful tool that can be applied to determine hydrogel dynamics.

SERS is a relatively common technology that greatly enhances the resolution of standard Raman spectroscopy [38]. This technique is generally of interest for potential uses in sensors. Its application depends on the presence of certain metals such as Au and Ag being present on the surface being analyzed, and thus, Ag particles have been included in a phenylalanine-based supramolecular hydrogel to be able to use SERS [39]. This technique was moreover used to characterize the weak intermolecular interactions involved in the formation of the hydrogel. In particular, changes in the Raman intensities as a function of temperature confirmed that the hydrogel was formed by intermolecular hydrogen bonding between carboxyl groups and oxalyl amide groups in the gelator molecules [39].

### 2.4. Circular Dichroism (CD)

**Figure 3:**
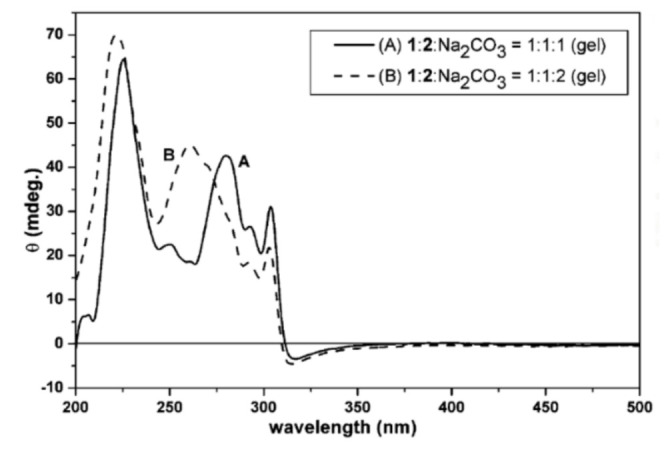
CD spectra of hydrogels prepared from *N*-fluorenylmethoxycarbonyl (Fmoc)-protected amino acids, NPC 15199 and a lysine derivative, with differing concentrations of Na_2_CO_3_. Reproduced with permission from [42] ©2004, Royal Society of Chemistry.

CD is a spectroscopic tool that works due to molecular interactions differing if exposed to either right or left chiral light, and it has long been used in biology to study the secondary structure of proteins [40,41]. By analyzing CD spectra, information about equilibrium constants, conformational changes, protein folding and denaturation, and the secondary structure of various macromolecules and their binding ligands can be gathered [40,41,42,43]. In an early application to supramolecular hydrogels, Yang et al. studied a potentially anti-inflammatory, novel agent made from a combination of two *N*-fluorenylmethoxycarbonyl (Fmoc) protected amino acids and used it to form self-assembled fibrous hydrogels, which allowed the integration of other drug molecules. With aims of investigating the structures of the molecules in the hydrogels and characterizing the hydrogels themselves, CD spectroscopy was employed to determine that π–π interactions existed between the various chemical groups. The peak at 221–224 nm was attributed to a helical conformation of the molecules, and peak shifts upon addition of Na_2_CO_3_ were attributed to the increased ionic strength as well as greater rigidity of the hydrogel (Figure 3) [42]. Thus, CD is a useful technique for understanding the structural conformations in supramolecular hydrogels, particularly for those based on amino acids or peptides.

## 3. Diffraction

While noting the unique behavior of light and its shape when being shone through a pinhole, scientist and researcher Francesco Grimaldi in 1618 termed the phenomenon “diffraction” [44]. Diffraction essentially involves taking a form of electromagnetic radiation, such as visible light or X-rays, and bouncing them off an object to produce a scattering effect that can reveal important characteristics of the material [45]. Thus, diffraction is used as a characterization technique for determining the crystal structure of materials, and the different types include XRD, neutron diffraction, and electron diffraction [46]. Since diffraction techniques work best for crystalline materials, this technique is used less frequently for the analysis of covalently-crosslinked polymer hydrogels, which often exhibit amorphous properties, but can be used for supramolecular hydrogels since their self-assembling nature often results in regions of crystallinity [47,48,49,50]. When applied to such supramolecular hydrogels, diffraction techniques assist in determining the arrangement and structures of molecules in the assemblies [51]. In this review, we focus on XRD because that is mainly what studies on supramolecular hydrogels have used for characterization, but we also mention neutron diffraction in the context of small-angle diffraction.

### 3.1. X-ray Diffraction (XRD)

**Figure 4:**
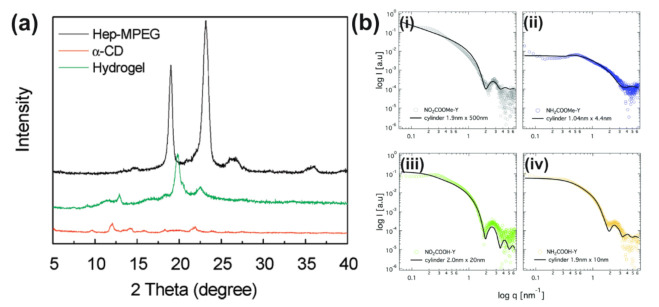
(**a**) X-ray diffraction (XRD) patterns of heparin-conjugated polyethylene glycol methyl ether (Hep-MPEG), α-cyclodextrin (α-CD), and a freeze-dried hydrogel composed of Hep-MPEG and α-CD. (**b**) Small-angle X-ray scattering (SAXS) data with curves fit to a cylindrical model for hydrogels made in 0.01 M NaCl from 3 wt% gelator: (i) NO_2_COOMe-Y, (ii) NH_2_COOMe-Y, (iii) NO_2_COOH-Y, and (iv) NH_2_COOH-Y. Reproduced with permission (**a**) from [48] ©2010, American Society of Chemistry; (**b**) from [17] ©2019, Royal Society of Chemistry.

Following Grimaldi’s understanding, scientist Wilhem Röntgen discovered the usage of X-rays while experimenting with discharge tubes in 1895, and, a few decades later, a group of three scientists performed successful XRD experiments in 1912 [44]. XRD is a non-destructive technique which relies on the phenomena of X-ray scattering to characterize and define various crystalline materials as the diffraction patterns display the atomic arrangements of the structure [45,46]. Powder diffraction is the most common type of XRD used to analyze solid state materials, and it can accommodate the use of samples in powder, thin film, or bulk form. Interestingly, XRD analysis has relied heavily on computational methods for data interpretation, including whole powder pattern fitting and solving crystal structures [44]. Thus, XRD is an excellent mechanism to aid in the investigation of crystalline materials in general, and most studies on supramolecular hydrogels have focused on this aspect. It is essential to fully understand and characterize the materials by their size, thickness, crystalline perfection, and shape [44,52]. For example, Ma et al. used XRD to characterize supramolecular hydrogels made from host–guest interactions between α-CD and heparin-conjugated poly(ethylene glycol) methyl ether (Hep-MPEG). The XRD spectra were compared for pure Hep-MPEG, pure α-CD, and the supramolecular hydrogel (Figure 4a). The hydrogel exhibited two XRD peaks not shown in either of the pure substances, which corresponded to the inclusion complexes formed from the crosslinking processes, thus verifying the supramolecular nature of the hydrogel [48]. More recently, Zhang et al. performed a study involving XRD used to characterize supramolecular hydrogels made from host–guest interactions between α-CD and monomethoxy poly(ethylene glycol) (PEG)-polycaprolactone (PCL) block co-polymer micelles as well as their loading with the drug diclofenac. Peaks from the drug were no longer present in the XRD spectra of the loaded hydrogels, suggesting that the drug was encapsulated in an amorphous state. Further, the hydrogels exhibited a peak on the XRD spectrum that was characteristic of inclusions formed by interactions between PEG and α-CD, allowing the researchers to conclude that these inclusions drove the formation of the supramolecular hydrogels [49], similar to the observations in the study by Ma et al. Finally, Zhu et al. used XRD to investigate how the addition of Au nanoparticles affected the crystallinity of a supramolecular gel made from 1,4,7,10-tetraazacyclododecane-1,4,7-triacetic acid and *N*-(4-aminobenzoyl)-L-glutamic acid diethyl ester in solvent mixtures of ethanol and water. Through the use of XRD on lyophilized samples, it was determined that the gel with the Au nanoparticles exhibited four additional peaks due to the crystal structures of the Au nanoparticles [50]. Thus, XRD is a useful characterization tool not only for supramolecular hydrogels but also for nanocomposite hydrogels due to their increased crystallinity.

It is also important to note that different types of XRD exist, such as wide-angle X-ray diffraction (WAXD) and small-angle X-ray scattering (SAXS), and these techniques have been increasingly used to study the structure of biological macromolecules [53]. For the characterization of inorganic materials, WAXD is used in order to determine the grain size and crystal phase of the material, while SAXD is useful for analyzing the surfaces inside the grains [54]. Both can be helpful characterization techniques for hydrogels, as discussed in the following subsections. The analysis of SAXS and WAXD data requires the use of computational methods for interpretation, and recent advances in simulation techniques such as molecular dynamics may help to address some of the limitations associated with low information content data as well as to improve predictions of structures and solvent scattering [55]. Moreover, new additions have been implemented to XRD, with synchrotron radiation sources proving to provide heightened levels of resolution and tunable radiation over more wavelengths [56].

#### 3.1.1. Wide-Angle X-ray Diffraction (WAXD)

WAXD has been used to study the structure of hydrogels [57,58,59,60], with László et al. using this technique to examine thermo-responsive poly(N-isopropylacrylamide) hydrogels. Using WAXD, local ordering of the polymer was able to be determined, forming a broad peak at approximately q = 0.56 Å^−1^. Moreover, in examining the absence of sharp diffraction lines in the WAXD region, the authors concluded that the polymer-laden phases of both the wet and dry polymer were amorphous [58]. Considering supramolecular materials, Li et al. examined a novel type of injectable and bioabsorbable hydrogel that was created from host–guest interactions between α-CD and poly(ethylene oxide)s (PEOs) [59]. Through the utilization of WAXD, the researchers were able to confirm the overall structure of the supramolecular hydrogels as well as the supramolecular self-assembly mechanisms to form them. Moreover, structural differences between inclusion complexes of α-CD and propionic acid versus α-CD and PEO as well as the specific threading of the α-CD onto the PEO chains were determined via WAXD [59]. Similarly, Zhang et al. performed WAXD on xerogels prepared from D-gluconic acetal-based derivative hydrogels to characterize the interactions between the gelator molecules and different Hofmeister salts. Analysis of the four main diffraction peaks suggested that the xerogel was composed of hexagonal cross packing with self-assembly driven by π-π stacking. The addition of the different salts caused the peaks to shift, supporting other methods that showed the properties of the hydrogels changed as a result of the different anions [60].

#### 3.1.2. Small-Angle X-ray Scattering (SAXS)

SAXS is a multi-purpose, universal research tool which aids in the real-time investigation of complex structures while being able to extract structural changes [17,61,62]. The proper visualization of the macromolecular size and shapes of various biological compounds is essential as these directly affect their function. SAXS offers a simple way to do this, increasing in use with the advancements of synchrotron X-ray detectors and sources; SAXS is commonly used to investigate supramolecular hydrogels. A few assumptions made in scattering techniques are that the particles used are identical, randomly oriented, and of sufficiently low concentration to prevent scattered wave interference and that a macromolecular solute and homogeneous solvent are used in the analyzed solution [17,61,62]. SAXS is compatible with various biophysical methods and can be used to define 3D structures to more than 15 Å resolution [61,62]. For example, Akkari et al. studied the supramolecular structure of poloxamer 407 (PL407) and poloxamer 188 (PL188) binary thermosensitive hydrogels, which formed micelles. SAXS confirmed their sol-gel transition from a lamellar structure at 25 °C to a hexagonal structure at 37 °C (Figure 4b). The researchers aimed to use these hydrogels to deliver the drug ropivacaine (RVC) to patients. Both scanning electron microscopy (SEM) and SAXS analyses displayed similar trends after the incorporation of RVC, with the hydrogel maintaining its supramolecular hexagonal structure [61]. Moreover, Drechsler et al. employed SAXS when investigating the formation of hydrogels and their structures derived from low-molecular-weight Y-shaped aromatic amide tetramers with different functional groups. The length scales associated with the gels’ self-assembly processes were analyzed using SAXS, and for three of the four compounds, the SAXS data fit with a cylindrical model. Using SAXS was essential in their experiment as it exposed shortcomings in their molecular dynamics simulations, which aimed at creating a model for molecular level aggregation, by showing an entirely different stacking mechanism forming the aggregates [17]. Thus, SAXS has proven to act as an effective tool to note shapes and structural changes during experimentation, and computational methods such as molecular dynamics provide a useful complement to this experimental technique.

### 3.2. Neutron Diffraction

Through a better understanding of neutrons, scientists have been able to use neutron diffraction to determine crystalline structures. Neutron diffraction is a type of elastic scattering technique in which there is a constant exchange of neutrons of similar energies. Although this tool is similar to that of XRD, the unique properties of neutron diffraction allow for the classification of materials that have similar elemental compositions while providing heightened analyses for the identification of a material in areas where XRD is lacking [63]. As with XRD, both small- and wide-angle neutron diffraction measurements can be obtained with the small-angle scattering, as described in the following subsection, being more prevalent [64].

#### Small-Angle Neutron Scattering (SANS)

Small-angle neutron scattering (SANS) is a scattering technique that probes material structures of various substances on the nanometer and micrometer scale, and it is particularly useful in analyzing supramolecular hydrogels [64,65]. For example, Mihajlovic et al. employed the use of SANS to better understand the micellar microstructure of hydrogels made from hydrophobic interactions. These hydrogels were composed of copolymers of PEG of differing molecular weights and dimer fatty acid (DFA) units. SANS analyses determined the aggregation number of the micelles, which ranged from 2 × 10^2^ to 6 × 10^2^ DFA units, and showed that this aggregation number increased with the molecular weight of the hydrophilic PEG block, with the quantitative information coming from fitting the experimental data to a theoretical computational model [65]. Similarly, SANS technologies, in combination with cryogenic transmission electron microscopy (cryo-TEM) were used to quantitatively analyze the microstructural characterization of micelles formed from a triblock copolymer called Pluronic F127. The scattering patterns were characterized at both low and high temperatures (range 5–95 °C), with a low absolute intensity and weakly dependent scattering function on the wave vector transfer, q, at low temperatures. However, as temperature increased, these characteristics changed, with a high absolute intensity and a stronger q dependence. These findings suggested that the copolymer aggregated at higher temperatures with increasing micelle size, which was also corroborated by the cryo-TEM analyses [66]. For the characterization of thermoreversible hydrogels, Lorson et al. also used SANS as, unlike conventional X-ray scattering, SANS provided an excellent level of contrast between the copolymer and solvent used as well as between the blocks of the copolymer. The thermogelling synthetic block copolymers were made of poly(2-methyl-2-oxazoline) and poly(2-n-propyl-2-oxazoline) and were proposed to potentially be useful in future tissue engineering applications. The fitting of the SANS data to a theoretical model revealed that the distinctive hydrogel had a bicontinuous, spongy structure. SANS and rheological techniques corroborated one another, reporting that the hydrogel’s structure was different from that of Pluronic hydrogels [67]. In another study, Wiener et al. used SANS to study the changes in structure for a tough, supramolecular hydrogel composed of random copolymers of *N*,*N*-dimethylacrylamide (DMA) and 2-(*N*-ethylperfluorooctane sulfonamido)ethyl acrylate (FOSA) by extrapolating correlations between the relaxation of stress and the network chains and nanodomains. Physical crosslinks were formed by the self-assembly of the FOSA units within the copolymers, with these nanodomains existing as hydrophobic aggregates. When the SANS analyses of the size, spacing, and anisotropy of the nanodomains were compared with stress-relaxation measurements, the authors were able to better understand the toughness of the supramolecular hydrogel on a molecular level [68]. This accurate analysis of structures of this scale is vital to the advancement of the field of materials science and engineering [64,65].

## 4. Microscopy

Microscopy is aimed at imaging materials at high resolution, from which structural and morphological details of materials can be seen and understood. Although microscopy can be used for the characterization of the morphology of many types of hydrogels as well as other materials, these techniques are particularly valuable for the characterization of supramolecular hydrogels because they allow for the visualization of the individual fibers of the self-assembled structures. The often nanoscale dimensions of these fibers also push the development of these techniques because they require imaging at higher and higher levels of resolution. Microscopy technologies that have been applied to supramolecular hydrogels include the broad categories of electron microscopy, SRM, and SPM.

### 4.1. Electron Microscopy

Electron microscopes utilize a beam of electrons to obtain high-resolution images of materials, although damage to the analyte can result. A vacuum is necessary to avoid interference of the beam, which also means samples must be dried before imaging. The most common electron microscopy techniques for hydrogel characterization are transmission electron microscopy (TEM) and SEM, with cryo-EM also frequently employed.

#### 4.1.1. Transmission Electron Microscopy (TEM)

**Figure 5:**
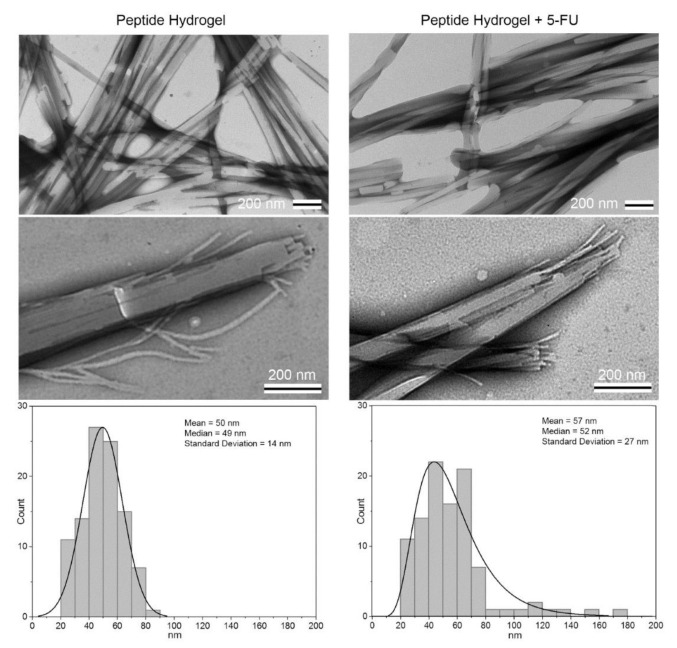
TEM micrographs and plots of fiber diameter measurements showcasing the morphological differences in heterochiral tripeptide hydrogels from the presence of the antineoplastic drug, 5-fluorouracil (5-FU). Reproduced under the terms of the Creative Commons Attribution License from [69]. ©2019 The Authors. Published by MDPI AG.

TEM relies on an electron beam passing through an ultra-thin sample to yield extremely high-resolution details on material structure and morphology. Samples often have to be sectioned into a thin form, although TEM gives details on internal structure as a result. Additionally, many hydrogel samples must first be prepared via negative staining in which the environment surrounding the hydrogel is stained rather than the hydrogel itself, outlining the hydrogel micro- and nanostructure with a compound that can provide contrast in the electron beam. In general, TEM is used to obtain high-resolution images of hydrogels. At this resolution, it becomes possible to observe fundamental properties of the hydrogel, including the size of individual components, such as fibrils or micelles, forming the hydrogel or their distribution in the hydrogel [42,69,70,71]. A comparison of hydrogels in different states is also useful. For example, Yang et al. employed TEM to conclude that amino acid-based hydrogels composed of NPC 15199 and a lysine derivative contained supramolecular assemblies of fibers made up of fibrils, which were approximately 11 nm in width. The higher resolution TEM images enabled the fibrils to be distinguished, whereas only the larger fibers could be observed in SEM images. After the addition of 5-fluoro-2′-deoxyuridine, the width of the fibrils demonstrated an increase to 20 nm [42]. Another example of TEM was given by Wang et al. who investigated a Gemini cationic surfactant and anionic aromatic compound multi-component hydrogel with the aim of constructing a drug delivery system that could carry multiple drugs [71]. TEM was used to image the hydrogel at the nanoscale, and the morphology of the hydrogel, encompassing the size of the fibrils (15–20 nm) and the way they had assembled, was observed. This information, along with evidence from several other characterization techniques, was used to support a proposed gelation mechanism involving packing of the aromatic groups and hydrophobic chains on opposite ends of a lamellar structure [71]. Additionally, in the area of drug delivery, Parisi et al. investigated a self-assembling peptide hydrogel based on leucine and phenylalanine and its effectiveness for the controlled release of 5-fluorouracil (5-FU), an antineoplastic drug. TEM was used to image the peptide hydrogel with and without the drug at the nanoscale, and morphological similarities and differences were observed, with the latter attributable to the addition of 5-FU (Figure 5). In particular, the diameter of individual fibrils that were formed by the peptide alone were larger than that of the diameter of those which included 5-FU. Moreover, both samples showed a similar assembly in terms of irregular twists and bundles. However, the hydrogel with 5-FU was more heterogeneous and had a wider size distribution [69].

#### 4.1.2. Scanning Electron Microscopy (SEM)

SEM uses an electron beam to scan the surface of a material. The technology does not require significant sample preparation for routine use, although it is less detailed and only provides surface imaging of materials. Despite their differences, SEM is often applied for similar reasons as TEM for imaging of supramolecular hydrogels. Changing the composition of a hydrogel and comparing it with the original by imaging both is a standard way of determining microscopic differences between the hydrogels. As an example, Tang et al. looked at a 1:1 guanosine–isoguanosine multi-component hydrogel assembled with aid from potassium ions using SEM for the purposes of drug delivery. Guanosine and isoguanosine single-component hydrogels were imaged alongside the multi-component hydrogel at the microscale. Potassium ions were also replaced with either lithium, sodium, rubidium, or cesium ions, which resulted in differences in the hydrogels’ structure, including size and shape. In the guanosine hydrogels, sheet-like structures were observed; in the isoguanosine hydrogels, porous, grass-like structures were found. Moreover, the guanosine hydrogel, which was induced with potassium ions, had structures with a width of 3 µm and a length of 200 µm; the isoguanosine hydrogel had a pore diameter of 1 µm. The 1:1 mixture of the two components formed porous, flower-like structures that had a diameter of 15 µm [72].

While SEM provides information on the surface structure of the hydrogel, focused ion beam (FIB)-SEM allows for 3D imaging. The surface layer is removed with the FIB, followed by a new image being taken; this process is repeated to build the 3D image [73]. FIB-SEM has been used to characterize extracellular matrix deposition by cells within hydrogels. For example, Olderøy et al. studied mesenchymal stem cells encapsulated in alginate hydrogels to further examine the assembly and distribution of the collagen synthesized by the cells. FIB-SEM was used to understand the distribution of collagen fibrils in a 3D network, which showed fibrils of 40–50 nm in diameter and several microns in length distributed in the image stack [73]. Thus, this application has excellent potential for the characterization of supramolecular hydrogels.

#### 4.1.3. Cryo-EM

**Figure 6:**
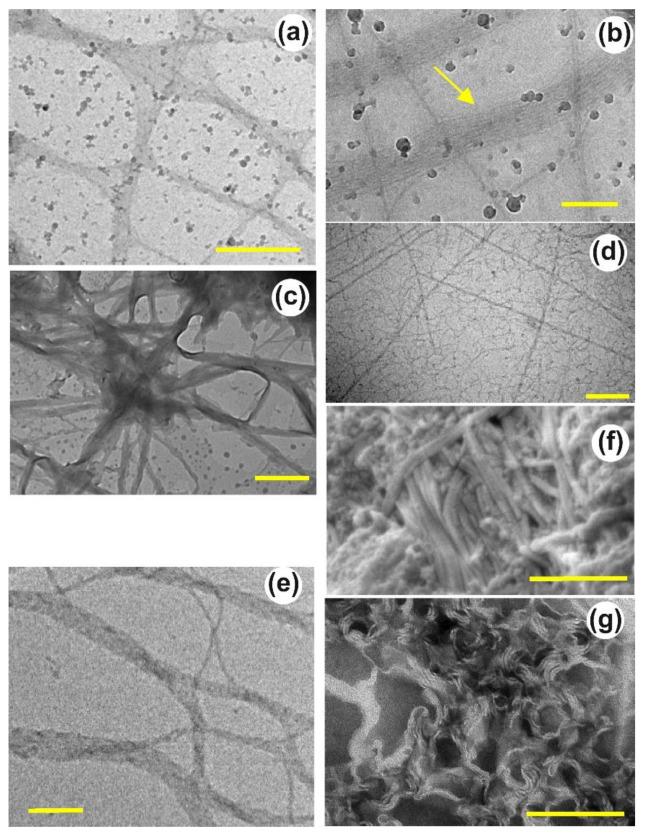
The morphologies of hydrogels formed from relatively concentrated solutions of a low-molecular-weight sugar-based hydrogelator with (**a–c**) showing ribbons and sheets, (**d**) showing micelles, and (**e–g**) showing helical fibers. (**a**), (**b**), (**d**), and (**e**) were acquired from cryogenic transmission electron microscopy (cryo-TEM), (**c**) and (**g**) from transmission electron microscopy (TEM), and (**f**) from cryogenic scanning electron microscopy (cryo-SEM). The bar indicates 200 nm in (**b**), (**d**), (**e**), and (**g**), 500 nm in (**a**) and (**f**), and 1 mm in (**c**). Reproduced with permission from [18] ©2017, Elsevier.

On the one hand, the radiation damage caused by the electron beam used in TEM and SEM can be limited by lessening the beam intensity, although this increases noise generated in the resulting image. On the other hand, high-resolution images with reduced signal-to-noise can be generated with stronger beams, although the damage to the sample cannot be ignored. This problem is addressed with cryo-EM, involving the rapid cooling of a sample and observing it in vitrified ice [74]. Cryo-EM also addresses many of the drying artifacts caused during sample preparation for traditional electron microscopy. Both TEM and SEM have variants that incorporate cryo-EM, with the cryogenic techniques providing significant advantages compared to their traditional TEM and SEM counterparts in terms of better revealing the morphological details of the specimen under investigation. These advantages are demonstrated in a study by Fitremann et al. who aimed to construct a low-molecular-weight sugar-based hydrogel with relatively simple synthesis steps to improve hydrogel reproducibility. TEM, cryo-TEM, and cryo-SEM were used to observe the hydrogel at varying gelator concentrations in solution to examine the micro- and nano-structures present in the sample at different steps during the synthesis process. TEM and the cryo-EM techniques gave size and shape details about the hydrogel that could be related to a gelation mechanism, which varied with concentration and preparation method, with visual differences between images from each microscopy technique (Figure 6). For example, for the 2 wt% hydrogel, which was prepared via shearing, big flexible sheets and twisted helical fibers were observed; both of these structures also had fibrous substructures, with the TEM, cryo-TEM, and cryo-SEM technologies providing information regarding spacing and fiber lengths to the nm [18].

A standard method for preparing samples for cryo-TEM is plunge-freezing, which involves submerging a thin film of a sample into a cryogen. However, one limitation for analyzing supramolecular materials, such as thermoreversible hydrogels formed by micelles of Pluronic F127, by cryo-EM is that samples of high viscosity are difficult to prepare with this method. Work on addressing this limitation has been performed in studies such as that of da Silva et al. By adjusting several parameters in the preparation procedure, such as the volume of solution used in the film and the temperature to which the sample is pre-cooled, the authors were able to obtain reproducible images of individual micelles as well as their structural evolution as they formed the hydrogels. They concluded that tuning the parameters in a systematic way could increase the number of materials able to be imaged via cryo-EM [75].

Cryo-FIB-SEM can also be applied to overcome some limitations in the preparation of hydrogels samples for traditional SEM imaging. Clarke et al. examined oligopeptide-based supramolecular hydrogels utilizing cryo-FIB-SEM. The pentapeptide sequences were composed of three isoleucine residues and two aspartic acid residues in different orders and were able to form hydrogels with varying degrees of stiffness. To prepare the samples, the hydrogels were placed in liquid ethane, followed by rapid freezing with the aim of avoiding drying artifacts by forming a thin layer of vitrified ice. A FIB of gallium ions created a cross-section in the sample, followed by a rise in temperature to sublimate the ice and better reveal the underlying structure. Cryo-FIB-SEM analyses showed that the peptide sequence influenced hydrogel microstructure with the hydrogels prepared from peptides of aspartic acid residues alternating with isoleucine residues formed nanofibers with a length many times longer than their respective width [76].

### 4.2. Fluorescence Microscopy

Fluorescence microscopy can also be used to investigate the structure of hydrogels. This type of microscopy relies on the material’s ability to fluoresce, where fluorescence deals with the absorption of light at one wavelength and its emission at another wavelength [77]. When proteins are labeled with a fluorescent molecule, high signal-to-noise ratios are obtained that enable the study of protein dynamics, such as adsorption on surfaces or folding and aggregation [78]. Similarly, the molecules that make up hydrogels can also be labeled with fluorophores to allow for imaging of hydrogel structure using fluorescence microscopy. A recent study conducted by Vandaele et al. investigated the structures of synthetic fibrous hydrogel networks prepared from oligo(ethylene glycol)-grafted polyisocyanides via fluorescence microscopy. The hydrogels were made of heterogeneous fibrous networks, and quantitative metrics, such as the size of the pores comprising these hydrogels, could be determined using image segmentation and computational analysis of the polymer network [79]. Fluorescence lifetime imaging microscopy (FLIM), which utilizes information from the time and frequency domains of the fluorescent signal instead of its intensity, has recently been applied in an interesting way to measure the local rheological properties of supramolecular hydrogels. Shi et al. studied the degradation of self-assembled peptide amphiphile nanofibers in response to the enzyme cathepsin B, and they were able to quantify the internal viscosity of the liquid phase of the nanofibers in the range of 200–800 cP by combining FLIM with BODIPY-C10, a molecular rotor that is environmentally sensitive [80]. Fluorescence microscopy is particularly relevant for the characterization of biomaterials because fluorescent probes and dyes are commonly used in cell and molecular biology research, thus providing complementary methodology to study cell-material interactions in situ. Further, fluorescence microscopy can often be performed on hydrogels in their hydrated state, avoiding issues associated with samples processing and drying artifacts. However, in the characterization of supramolecular hydrogels, which are often hierarchical structures with some features on the nanoscale, the resolution of standard fluorescence microscopy techniques is often insufficient.

#### Super-Resolution Microscopy (SRM)

**Figure 7:**
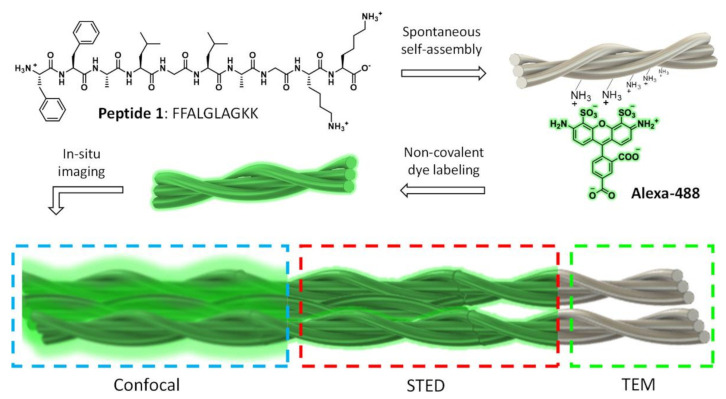
Schematics illustrating three different characterization methods for peptide-based nanostructures, including non-covalent labeling with a fluorescent dye (Alexa-488): confocal (fluorescence) microscopy, stimulated emission depletion (STED), and transmission electron microscopy (TEM) with different levels of resolution for the nanofibers. Reproduced with permission from [82] ©2020, American Society of Chemistry.

One relatively recent advancement in materials characterization techniques is super-resolution microscopy (SRM), which is not restricted by the diffraction limit, and thus can achieve better resolution than with traditional fluorescence microscopy techniques [81,82,83]. SRM is minimally invasive to the sample, especially when compared with electron microscopy techniques, and it can be used for studying biomaterials in situ, with potential application to supramolecular hydrogels. Additionally, SRM characterization is useful for analyzing the 3D molecular structure of a material, with the best resolution at approximately 5 nm, and it can generate multi-color images when coupled with fluorescent labelling, making SRM a useful technique for analyzing complex materials with multiple components [81,83]. While SRM offers these many advantages over traditional characterization methods, this technology does have limitations, such as not being able to be used with bulk materials that are thick or opaque [83]. The main types of SRM include single-molecule localization microscopy (SMLM), structured illumination microscopy (SIM), and stimulated emission depletion (STED) [81,83]. SMLM provides the best resolution, but it requires the use of certain probes and buffers to provide optimal conditions for imaging that may damage living cells, and thus this technique is best suited for non-living samples that require precise resolution. For SIM, less light is required for imaging, making it ideal for samples that may be sensitive to light, but it has the least precise resolution out of the three techniques. STED has resolution better than SIM but worse than SMLM, but it is easier to prepare the sample than SMLM, making it a good intermediate choice. Depending on the method used, varying amounts of computational post-processing are required to generate the images [81]. A recent study by Kumar et al. investigated the use of STED imaging for supramolecular nanostructures made from self-assembling peptides to capture images that had resolution of less than 60 nm (Figure 7). They also used this technique, along with non-covalent fluorescent labeling, for imaging of the nanomaterial during a dynamic enzymatic degradation process, thus making STED a promising method for studying moving molecules at a small scale. Through the STED imaging technique of the dynamic process, the degradation of the nanostructure’s fibers could be determined over time, and the researchers found that the inner fibers began degrading before the exterior ones. However, they also noted that STED images had a lower resolution when measuring the dynamic process than when used with static materials due to the exposure time of two images per minute adding blurriness to the image [82].

### 4.3. Scanning Probe Microscopy (SPM)

SPM describes a set of microscopy techniques that employ a physical probe that scans over the material surface to generate a high-resolution image. In the context of supramolecular hydrogels, AFM, also called scanning force microscopy, is the most well-known scanning probe microscopy technique, although others include scanning tunneling microscopy [84] and scanning near-field optical microscopy [85,86]. In this review, we focus on AFM because that is the most widely used technique for the characterization of supramolecular hydrogels.

#### Atomic Force Microscopy (AFM)

**Figure 8:**
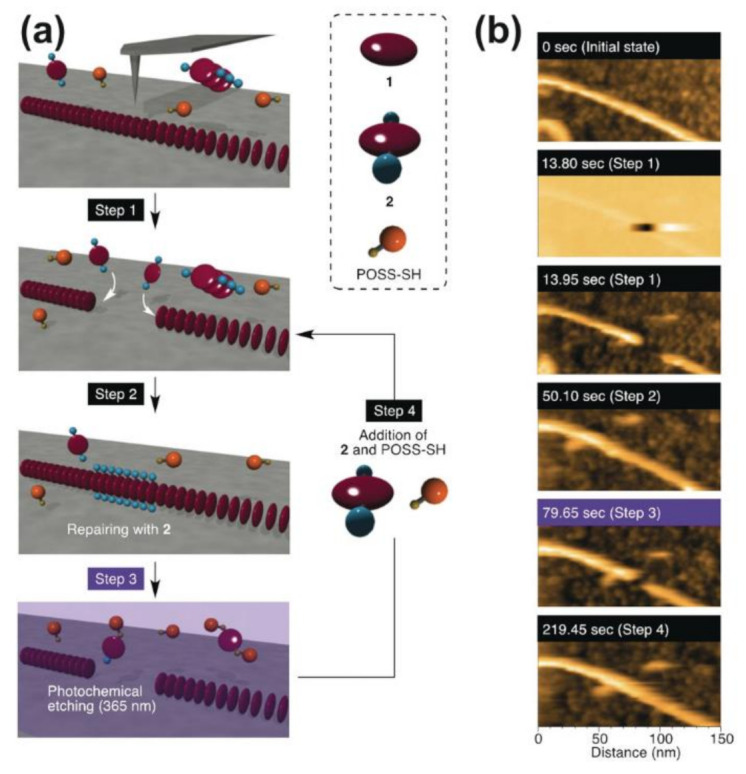
(**a**) Schematic of the manipulation of a segment of the porphyrin-based supramolecular polymer using the atomic force microscopy (AFM) probe. (**b**) AFM images from that process. Reproduced with permission from [89] ©2018, Wiley-VCH Verlag GmbH & Co. KGaA.

AFM is a useful technique that uses a probe that physically moves over the surface of the sample to generate an image and obtain information about the sample’s surface. Based on the size of the probe, AFM can be used at different scales, between a few nanometers and several hundred micrometers, making it a versatile technology that can analyze many different types of materials, including biomaterials [87]. AFM has been used in several studies to analyze supramolecular materials, and it is a promising technique for future advancements in materials science characterization [88,89,90]. For instance, a study by Roy et al. used AFM to analyze the structure of Fmoc-peptide-based supramolecular hydrogels and study how their self-assembled structure changed when adding different salts. Adding phosphate ions resulted in hydrogels composed of dense bundles of fibers, while the addition of thiocyanate ions created aggregates with spherical shapes [88]. The dimensions of these structures can also be extracted from the AFM images. For example, AFM was used by EzEldeen et al. to take images of a supramolecular hydrogel made from the self-assembling peptide RADA16. Besides qualitatively showing the presence of nanofibers, the AFM images were analyzed by a computational fiber extraction algorithm, which gave numerical data on the dimensions, straightness, roughness, and alignment of the fibers that made up the microstructure of the hydrogels [91]. Additionally, Fukui et al. used high-speed AFM for imaging dynamic processes of supramolecular polymers made from a derivative of porphyrin, such as self-repair and growth of the polymer chains. Due to the probe physically interacting with the surface of the material, they were also able to use AFM to interact with the polymer and add functional groups to the surface. For example, a segment of one supramolecular polymer could be removed and replaced with a segment of another composition to generate a copolymer (Figure 8) [89].

It is important to note that AFM can be used for both imaging and determining the mechanical properties of a material. The mechanical properties of biomaterials are very important for their function. For example, substrate elasticity has been shown to have an effect on stem cell lineage specification, with soft matrices promoting neurogenic differentiation [92]. Thus, methods for the characterization of soft materials using AFM are relevant [93,94], and this technique has been applied to supramolecular hydrogels. For example, Alakpa et al. created supramolecular amino acid-based hydrogels using Fmoc-serine and Fmoc-diphenylalanine, and they manipulated the stiffness of the material to optimize stem-cell differentiation for potential application to regenerative medicine. Using AFM, they captured images of the materials and also generated force-indentation curves to obtain stiffness measurements. They were successfully able to synthesize hydrogels at three different stiffness levels by varying the composition, which is then influenced the differentiation of stem cells to the neuronal, chondrogenic, and osteogenic lineages, respectively [90]. Thus, AFM is a versatile characterization tool and holds promise for future studies of supramolecular hydrogels due to its high resolution imaging and ability to measure mechanical properties and manipulate materials.

## 5. Mechanical and Surface Characterization

Mechanical and surface characterization of supramolecular hydrogels includes studying their rheological, stretching, and lubrication properties. Rheological characterization of mechanical properties at both the macro- and microscale is important for all hydrogels, but it is specifically relevant for supramolecular hydrogels due to their heightened ability for shear-thinning and self-healing behavior, which results from their self-assembled structures. In contrast to covalently crosslinked hydrogels, those that are formed from non-covalent interactions are also more likely to demonstrate stress relaxation. While the weak non-covalent interactions forming supramolecular hydrogels tend to lead to poor mechanical properties, these materials can provide an improvement in tensile properties, including recovery after stretching, when they are used as a part of multi-component interpenetrating network hydrogels. The surface properties of supramolecular hydrogels can be characterized by techniques such as DLS, to measure the zeta potential of self-assembled structures, as well as tribology to measure lubrication. Finally, this section also briefly covers computational methods for studying rheological properties of supramolecular hydrogels, which is a promising area for future research.

### 5.1. Rheology

Another interesting way to study supramolecular hydrogels is through rheology. Rheology provides information on the mechanical properties of the hydrogel, such as the storage modulus, as well as the degree of flow for hydrogel materials, especially when under shear stress. The latter is particularly useful when considering these materials for potential application to 3D printing or bioprinting where the bioink needs to flow through a nozzle and then solidify into a 3D structure [67,95]. Rheological properties of shear-thinning and self-healing are also important for the processing of hydrogels for tissue engineering and drug delivery applications as well as for evaluating their potential for minimally invasive delivery in vivo via injection [95,96]. Through measurement of the storage modulus, the stiffness of the hydrogel can be determined at the bulk level, providing a complementary methodology to AFM techniques [97,98].

The rheological properties of a hydrogel are highly dependent on the mechanism and degree of crosslinking as well as the structure of the molecules, and these properties can be tuned by changing the structure, molecular weight, or concentration of the gelator as well as by adding other molecules into the hydrogel network [60,67]. Moreover, rheological characterization is a relatively fast process that requires only a small amount of hydrogel sample [96]. A commonly used technique for rheological characterization in the linear-viscoelastic regime (LVE) of the material is small-amplitude oscillatory shear (SAOS), and this includes the use of frequency sweep tests, time sweep tests, and strain sweep tests. SAOS is a non-destructive technique that involves putting a small amount of the material between two plates on the rheometer so it can be deformed by a torsional oscillator at a small amplitude. This is useful for determining rheological parameters such as the shear modulus *G*, the storage modulus *G*′, and the loss modulus *G*″, and SAOS can also be used to determine the gelation time [96,99]. Further, a non-linear technique outside of the LVE involves large-amplitude oscillatory shear (LAOS), and it is recommended to perform this after completing SAOS because non-linear characterization can irreversibly damage or disrupt the hydrogel. A study by Lorson et al. investigated the rheology of supramolecular hydrogels that had thermogelling properties, making them ideal for bioprinting applications [67]. By studying a supramolecular hydrogel composed of a 20 wt.% methyl-P[nPrOzi_50_-b-MeOx_50_]-piperidine-4-carboxylic acid ethyl ester polymer, the researchers found a *G*′ value of 4kPa, indicating relatively high mechanical strength for a supramolecular hydrogel [67].

### 5.2. Shear-Thinning and Self-Healing Characterization

**Figure 9:**
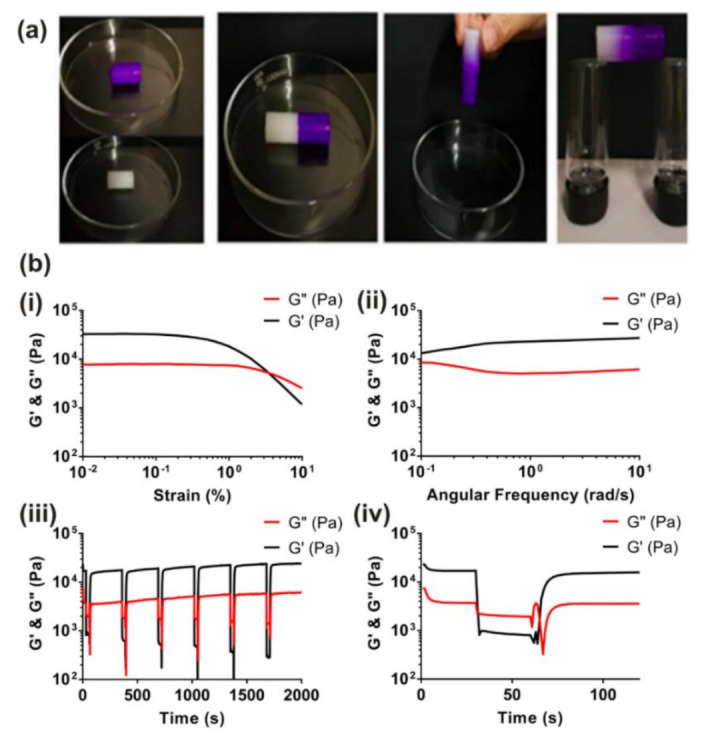
(**a**) Illustration of the self-healing properties of a 2.0% w/v hydrogel formed from a d-gluconic acetal-based derivative using hydrogel pieces with and without methylene blue dye. (**b**) Rheological properties of a shear-thinning and self-healing supramolecular hydrogel based on a bis-urea derivative, showing (i) a strain sweep, (ii) a frequency sweep, and (iii, iv) dynamic strain amplitude tests. Reproduced (**a**) under the terms of the Creative Commons CC BY License from [60] ©2019 The Authors; published by Springer Nature and (**b**) with permission from [14] ©2019, Royal Society of Chemistry.

Both shear-thinning and self-healing are important rheological (thixotropic) properties for hydrogels to exhibit for improved application to 3D printing, tissue engineering, and drug delivery [67,95,96,100]. When some supramolecular hydrogels are placed under shear stress, shear-thinning occurs, and it results in a decrease in viscosity of the material. Self-healing is the opposite process, occurring when the shear stress is released and the viscosity increases again [100]. Self-healing can be demonstrated visually by the formation of a continuous hydrogel from separate pieces (Figure 9a) [60,101], and shear-thinning and self-healing behavior is usually characterized by rheology where the *G*″ is above the *G*′ with higher shear, indicating disruption of the hydrogel network, and *G*′ returns to its original level after the reduction in shear (Figure 9b) [14,101]. These cycles of shear-thinning and self-healing can also be repeated many times with no degradation of the *G*′ of the hydrogels. The supramolecular hydrogels composed of a methyl-P[nPrOzi_50_-b-MeOx_50_]-piperidine-4-carboxylic acid ethyl ester polymer described above also exhibited favorable shear-thinning and self-healing properties, indicating that it was viable for bioprinting applications. When exposing the hydrogels to a cycle of a low amplitude strain, then a higher strain, followed by a return to the low strain level, the hydrogel recovered almost immediately when returning to the low strain and its *G*′ value was comparable to before the high strain was applied. This fast recovery indicated that the material had favorable self-healing properties [67]. Supramolecular hydrogels exhibiting host–guest interactions can also exhibit these properties, as shown in a study using β-cyclodextrin (β-CD) and adamantane as the host and guest, respectively. These supramolecular hydrogels underwent shear thinning when the host–guest complexes disassembled, and self-healing occurred when the complexes reassembled afterwards. However, this reversibility was also a disadvantage for the bulk mechanical strength of the material, and so Loebel et al. addressed this issue by integrating the host–guest complexes in a network of hyaluronic acid polymers that could be covalently bound together to reinforce the hydrogel in a secondary crosslinking step, thus improving the mechanical properties while maintaining the shear-thinning and self-healing properties [95]. Other studies have performed rheological characterization of supramolecular hydrogels to evaluate their shear-thinning and self-healing behavior. A study by Zhang et al. focused on tuning the rheological characteristics of supramolecular hydrogels made from a d-gluconic acetal-based derivative that exhibited self-healing properties. They found that by varying the amount of Hofmeister salts added to the hydrogels, this could impact the thixotropic properties of the hydrogels, which could make the material either more or less viscous under shear stress depending on the particular salt added. This is an important finding because, by altering the thixotropic and rheological properties of a hydrogel, its material properties can be optimized for bioprinting applications [60].

### 5.3. Micro-rheology

Micro-rheology involves smaller-scale measurements than traditional shear rheology techniques, such as those mentioned in the previous sections. Because of this, micro-rheology has the advantages of requiring less sample material than macro-rheology as well as having higher sensitivity by using higher frequencies for measurements [102,103]. Examples of micro-rheological characterization techniques involve particle tracking measurements using fluorescence microscopy techniques such as FLIM, which were already mentioned in Section 4.2, as well as DLS and DWS. DLS involves shining light at a sample and tracking the scattering motion of the light from micro- or nanoparticles in the material over time to determine viscosity and *G*′ values, which can be used to characterize the gel point of a supramolecular hydrogel [104]. However, DLS only works well on transparent materials, and thus DWS is an alternative technique for micro-rheology measurements in opaque materials [105]. In a study by Frith et al., DLS was used to investigate the micro-rheology of supramolecular hydrogels made from Fmoc-tyrosine. In order to use DLS, tracer particles made from polystyrene were added to the hydrogel at the maximum concentration that did not interfere with proper DLS measurements. The maximum concentration of 0.001% w/w was only viable for the first 240 min before supramolecular structures formed and blocked the DLS measurements. Interestingly, the results obtained using micro-rheology were different from those measured with bulk rheology, and further, the micro-rheology measurements were dependent on the particle size of the probes used [104]. Additionally, Ozaki et al. used both DLS and DWS to characterize supramolecular hydrogels made from a polyacrylamide derivative that was crosslinked with nickel ions. Using DWS, it was determined that the addition of nickel ions increased the viscoelasticity of the hydrogel and that the gel point was at a nickel concentration of 1.1 mM. With DLS, the researchers used an autocorrelation function to characterize the intensity of the scattered light and to classify particle movement during gelation by the characteristic time for diffusion. Without the nickel ions, the hydrogel exhibited two different modes, either a faster collective diffusion or a slower diffusion of clusters of particles. However, with nickel added, there was an additional intermediate mode, and it was concluded that this was due to the crosslinking from the nickel ions [104]. Both studies also used macro-rheology to further characterize the hydrogels, and the combination of micro- and macro-rheological characterization can be helpful for understanding the properties of supramolecular hydrogels [104,106]. Additionally, it is important to note that DLS can be used for characterization of the size and surface charge of particles within a supramolecular hydrogel, as shown in several studies [107,108,109].

### 5.4. Other Mechanical Testing

Another important mechanical property for supramolecular hydrogels lies in their stretching ability in order to mimic the properties of natural tissues for applications to tissue engineering and other biomedical purposes; this can be evaluated via tensile testing [110,111]. The dynamic nature of the non-covalent interactions forming supramolecular hydrogels often provides the advantage that these materials can recover after stretching. For example, Kakuta et al. prepared a tough, elastic supramolecular hydrogel from interactions between β-CD acrylamide (host molecule) and adamantine acrylamide (guest molecule). In tensile testing, the β-CD-adamantane crosslinked poly(acrylamide) hydrogel demonstrated a 990% stretching ability. Moreover, after being stretched to 180%, the hydrogel was able to recover its original morphology. Contrastingly, chemically crosslinked poly(acrylamide) hydrogels did not recover and did break when tested in tension [110]. Similarly, Le et al. prepared a poly(acrylamide) hydrogel that displayed triple shape memory features by using dynamic phenylboronic-diol ester bonds and alginate crosslinked with calcium ions. By incorporating double network structures, the tensile strength of the hydrogel improved, with one formulation being able to self-heal and recover back to its initial state after being stretched by over 1200%. These self-healing properties were confirmed using a combination of rheological analyses and the manual tensile test [111].

### 5.5. Lubrication Characterization

Tribology studies the friction, lubrication, and wear in tribosystems, which consist of surfaces in physical contact along with the factors affecting the friction and wear behavior between them [112]. Articular cartilage, a significant biological tribosystem, has been demonstrated to reduce friction and wear via boundary lubrication, which relies on a film on the cartilage surface, and biphasic lubrication, in which pressurized interstitial fluid reduces the load on the solid contact surface. Owing to their similar tribological properties, hydrogels are often considered when designing and selecting materials to imitate biological tribosystems such as cartilage for biomedical purposes [113,114,115]. For these reasons, understanding lubrication and friction behavior is vital, and tribometers are tools that have been designed to measure such properties. They load a mass, often a ball, onto the surface being investigated before applying some force, such as a shear or linear sliding force, to measure tribological parameters such as the coefficient of friction experienced between the surfaces, and this technique has been applied to hydrogels generally and supramolecular hydrogels in particular [115,116,117]. To illustrate, Lin et al. added one of the phosphatidylcholine lipids to hydrogels made from poly (hydroxyethyl methacrylate), polymethacrylamide, poly(hydroxyethyl methacrylate-co-methacrylic acid), poly(acrylic acid-co-dimethacrylamide), and crosslinked gelatin methacrylate. Using a Universal Mechanical Tester tribometer in several reciprocating friction tests, the coefficient of friction between the hydrogels and a sliding steel sphere was determined at multiple temperatures, load, and sliding speeds. The addition of the lipids was found to significantly and consistently reduce friction because of the constant availability of the lipids at the hydrogel’s boundary layer [117]. Meanwhile, Milner et al. designed a triple network hydrogel by incorporating the lubricant poly(2-methacryloyloxyethyl phosphorylcholine) (PMPC) into a double network hydrogel composed of poly(2-acrylamido-2-methylpropane sulfonic acid) and poly(acrylamide). A tribometer was used to conduct a reciprocating friction test to determine the sliding friction properties of the double network and PMPC triple network hydrogels for several sliding speeds of a CoCr alloy contact ball. The addition of PMPC reduced the coefficient of sliding friction, making the triple network hydrogel more effective for partial joining repair. Combined with other characterization techniques, it was found that the triple network hydrogel was able to mimic both biphasic and boundary lubrication mechanisms [116]. Overall, tribometers are useful tools in analyzing friction and lubrication for supramolecular hydrogels and can be considered when such properties are a focus of study.

### 5.6. Computational Modeling of Rheological Properties

A promising approach to complement the experimental rheological characterization of hydrogels is the use of modeling simulations, which can help to reduce the time and costs associated with the experiments. This approach is feasible because the molecular structure of the hydrogel network influences its rheological properties, and increasingly sophisticated computational models of supramolecular materials are being developed, as reviewed by Van Lommel et al. [118]. That review provides further detail regarding computational modeling techniques, including DFT, molecular dynamics calculations, and crystal structure prediction, which have been applied to understand many aspects of supramolecular gels [118]. These computational methods can assist in determining rheological properties of supramolecular hydrogels. For example, Tang et al. used a numerical computer model based on Monte Carlo simulations to create a network of polymer chains that could be analyzed to predict rheological properties, such as network percolation and local defects and their relation to the shear modulus G [119]. Another study was performed by Drozdov and Christiansen in which supramolecular hydrogels in LVE were computationally modeled to predict oscillatory *G*′ and *G*″ values under varying conditions of temperature, pH, and concentration. The researchers determined that the simple linear viscoelastic model, which also addressed limitations with conventional Maxwell-type models, could accurately predict experimental shear oscillation results for crosslinked hydrogels made of proteins, poly(acrylamide-acrylic acid), PMPC, or PEG. These hydrogels cover a range of chemical structures and crosslinking mechanisms and show the broad applicability of the technique [120]. Thus, modeling simulations are a useful tool for facilitating the characterization of material properties and should be considered for future studies.

## 6. Conclusions

Supramolecular hydrogels are gaining in importance for several applications, and thus it is important for proper characterization of their material properties. In this review, we compiled a comprehensive report of current characterization methods as well as promising techniques for future studies. For spectroscopy, methods include NMR, absorption spectroscopy, Raman spectroscopy, and CD, and for diffraction, the most widely-used technique is XRD, although other methods such as neutron diffraction also can be beneficial for characterization. Small-angle scattering analyses are particularly interesting for gaining insight into the mechanism of self-assembly and the structure of supramolecular hydrogels. Traditional electron microscopy methods include TEM and SEM, with cryo-EM and FIB-SEM having the potential to improve the characterization of supramolecular hydrogels by reducing preparation artifacts in future studies. Additionally, SRM and SPM are promising techniques for higher resolution imaging. AFM offers the potential to measure mechanical properties such as stiffness by having the probe physically interact with the material in addition to image its topography. Furthermore, rheological characterization is important for supramolecular hydrogels to determine the mechanical properties of the hydrogels, and additional methods to characterize mechanical and surface properties include micro-rheology, DLS, tensile testing, and tribology. Future research should take advantage of these promising characterization techniques in order to better understand the properties of supramolecular hydrogels and their applications to the fields of biomaterials, energy, optoelectronics, and water purification. Additionally, as computational methods in data processing and analysis become more powerful and widely-used, resulting in more precise interpretation of increasingly higher resolution images, we suggest utilizing this methodology to complement the experimental characterization of supramolecular hydrogels in future studies.

## Data Availability

Not applicable.

## Abbreviations

α-CD	α-cyclodextrin
AFM	Atomic force microscopy
ATR	Attenuated total reflection
β-CD	β-cyclodextrin
CD	Circular dichroism
cryo-EM	Cryogenic electron microscopy
cryo-TEM	Cryogenic transmission electron microscopy
Dex	Dexamethasone sodium phosphate
DFA	Dimer fatty acid
DFT	Density functional theory
DLS	Dynamic light scattering
DMA	*N,N*-dimethylacrylamide
DWS	Diffusing-wave spectroscopy
Fmoc	*N*-fluorenylmethoxycarbonyl
FIB	Focused ion beam
FLIM	Fluorescence lifetime imaging microscopy
FT-IR	Fourier-transform infrared
FOSA	2-(N-ethylperfluorooctane sulfonamido)ethyl acrylate
Hep-MPEG	Heparin-conjugated poly(ethylene glycol) methyl ether
LAOS	Large-amplitude oscillatory shear
LVE	Linear-viscoelastic regime
MAS	Magic-angle spinning
NMR	Nuclear magnetic resonance
PCL	Polycaprolactone
PEG	Poly(ethylene glycol)
PEO	Poly(ethylene oxide)
PMPC	Poly(2-methacryloyloxyethyl phosphorylcholine)
PL188	Poloxamer 188
PL407	Poloxamer 407
RQC	Residual quadrupolar coupling
RVC	Ropivacaine
SANS	Small-angle neutron scattering
SAOS	Small-amplitude oscillatory shear
SAXS	Small-angle X-ray scattering
SEM	Scanning electron microscopy
SERS	Surface-enhanced Raman scattering
SIM	Structured illumination microscopy
SMLM	Single-molecule localization microscopy
SPM	Scanning probe microscopy
SRM	Super-resolution microscopy
STD	Saturation transfer difference
STED	Stimulated emission depletion
TEM	Transmission electron microscopy
UV–vis	Ultraviolet–visible
WAXD	Wide-angle X-ray diffraction
XRD	X-ray diffraction
3D	Three-dimensional
5-FU	5-fluorouracil
